# Hermansky-Pudlak Syndrome and Lung Disease: Pathogenesis and Therapeutics

**DOI:** 10.3389/fphar.2021.644671

**Published:** 2021-03-18

**Authors:** Pamela Velázquez-Díaz, Erika Nakajima, Parand Sorkhdini, Ashley Hernandez-Gutierrez, Adam Eberle, Dongqin Yang, Yang Zhou

**Affiliations:** ^1^ Department of Biology, University of Puerto Rico-Humacao, Humacao, Puerto Rico; ^2^ Department of Molecular Microbiology and Immunology, Brown University, Providence, RI, United States

**Keywords:** hermansky-pudlak syndrome, pulmonary fibrosis, lung, therapeutics, immunopathogenesis

## Abstract

Hermansky-Pudlak Syndrome (HPS) is a rare, genetic, multisystem disorder characterized by oculocutaneous albinism (OCA), bleeding diathesis, immunodeficiency, granulomatous colitis, and pulmonary fibrosis. HPS pulmonary fibrosis (HPS-PF) occurs in 100% of patients with subtype HPS-1 and has a similar presentation to idiopathic pulmonary fibrosis. Upon onset, individuals with HPS-PF have approximately 3 years before experiencing signs of respiratory failure and eventual death. This review aims to summarize current research on HPS along with its associated pulmonary fibrosis and its implications for the development of novel treatments. We will discuss the genetic basis of the disease, its epidemiology, and current therapeutic and clinical management strategies. We continue to review the cellular processes leading to the development of HPS-PF in alveolar epithelial cells, lymphocytes, mast cells, and fibrocytes, along with the molecular mechanisms that contribute to its pathogenesis and may be targeted in the treatment of HPS-PF. Finally, we will discuss emerging new cellular and molecular approaches for studying HPS, including lentiviral-mediated gene transfer, induced pluripotent stem cells (iPSCs), organoid and 3D-modelling, and CRISPR/Cas9-based gene editing approaches.

## Introduction

Hermansky-Pudlak Syndrome (HPS) is a rare, autosomal recessive, multisystem disorder that has a disproportionately high effect on Puerto Ricans (1 in 1800) (Witkop et al., 1990). This disorder is caused by genetic mutations which result in defective lysosome-related organelles (LROs) such as melanosomes, which synthesize and store melanin, and platelet dense granules, which store small signaling molecules involved in platelet aggregation (Hermansky and Pudlak 1959; Seiji et al., 1963; King and Reed, 2002). Consequently, patients with HPS often develop oculocutaneous albinism (OCA), which results in hypopigmentation of hair, skin, and eyes, iris transilluminations, visual acuity, congenital nystagmus, foveal hypoplasia, and increased optic nerve decussation (Gahl et al., 1998; Power et al., 2019). Other symptoms include bleeding diathesis, and in a large number of individuals, immunodeficiency, granulomatous colitis, and pulmonary fibrosis. Affected individuals with bleeding diathesis may experience variable bruising, gingival bleeding, postpartum hemorrhage, colonic bleeding, and epistaxis (Huizing et al., 1993). Granulomatous colitis, which affects approximately 15% of patients with HPS, has similar clinical and pathological presentations as chronic ulcerative colitis and Crohn’s disease (Huizing et al., 1993; Grucela et al., 2006; Hazzan et al., 2006; Salvaggio et al., 2014).

HPS pulmonary fibrosis (HPS-PF) is a highly penetrant pulmonary fibrosis that occurs in patients with subtypes HPS-1, HPS-2, and HPS-4 (White et al., 1984; Brantly et al., 2000; Bachli et al., 2004). Onset usually occurs at 30–40 or 50–60 years of age, depending on the individual’s genetic makeup and response to inflammation (Vicary et al., 2016). HPS-PF shares a similar histological pattern as idiopathic pulmonary fibrosis (IPF) and is characterized by the development of dyspnea and incrementing debilitating hypoxemia (American Thoracic Society, 2002; Vicary et al., 2016). Much like IPF, HPS-PF results in progressive and irreversible scarring of lung tissue that ultimately leads to respiratory failure and death within approximately 10 years of HPS-PF onset (American Thoracic Society, 2002; Gahl et al., 2002). There are currently no available therapeutic interventions designed to treat HPS-PF, and the mainstay of clinical management is lung transplantation. However, the processes of finding a donor and performing the transplant can be difficult and risky. In Puerto Rico, for example, centers capable of performing lung transplantation are not available (Vicary et al., 2016). Thus, there is a pressing need to better understand the underlying mechanisms and pathogenesis of HPS and HPS-PF in order to begin developing effective therapeutic treatments.

Cellular processes that contribute to the pathogenesis of HPS-PF include apoptosis and dysfunction of type II alveolar (AT2) cells, and immune cell activation and dysfunction leading to alveolar inflammation (Fontana et al., 2006; Phan 2012; Trimble et al., 2014; Gil-Krzewska et al., 2017). Clinical studies suggest that mast cells and fibrocytes also likely play a role in HPS-PF although their mechanism of action is not fully understood (Trimble et al., 2014; Kirshenbaum et al., 2016). Studies using murine models of HPS have shined a light on the molecular mechanisms of fibroprolifeation in HPS. High levels of Chitinase-3-like 1 (CHI3L1), a prototypic chitinase-like protein, have been associated with tissue injury and remodeling in various forms of pulmonary fibrosis (PF), including HPS-PF (Zhou et al., 2015). Galectin-3 (Gal-3), a β-galactoside-binding lectin that interacts with CHI3L1 and its receptor IL-13Rα2, is associated with the progression of HPS-PF (Zhou et al., 2018). Additionally, various matrix metalloproteinases (MMPs) that have been shown to contribute to other forms of PF, is dysregulated in the HPS lung (Summer et al., 2019). Defective autophagy may also play a role in the development of HPS-PF (Ahuja et al., 2016). While many of these cellular and molecular processes have been identified as important contributors to the development of HPS-PF, little is known about the mechanisms linking these processes to the actual fibroproliferative processes, and no therapeutic has been developed to target these cell and molecular pathways.

The goal of this review is to summarize the current understanding of HPS, focusing on its genetic basis, epidemiology, and current clinical management, along with the cellular and molecular pathways involved in the progression of HPS-PF. This review will also discuss novel models for future studies of HPS-PF, potential targets for treatment, and areas of research within HPS that require further investigation.

## Genetics

**FIGURE 1:**
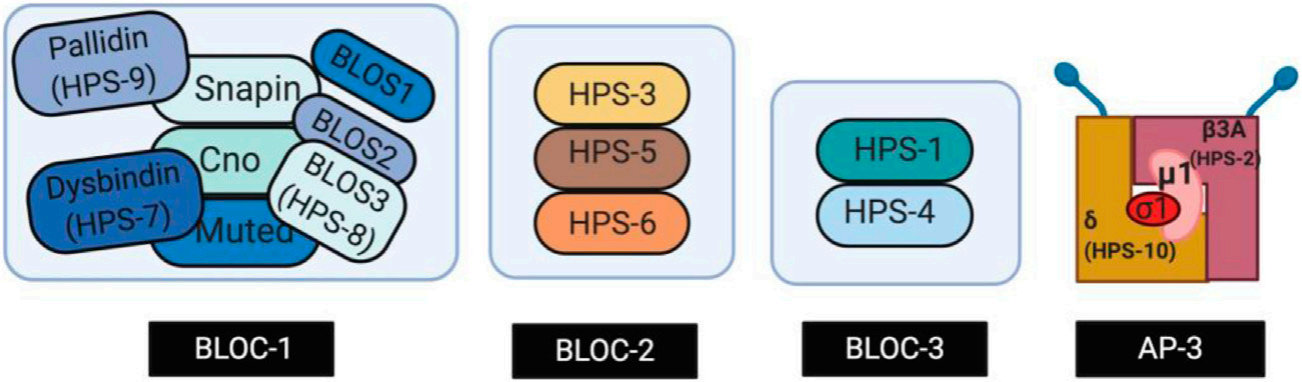
Genes encoding subunits of four proteins complexes called the Biogenesis of Lysosome-related Organelles Complexes (BLOC-1, -2, -3) and Adaptor Protein-3 (AP-3). The protein complexes play a role in the intracellular trafficking required for LRO biogenesis. Created with BioRender.com.

The ten subtypes of HPS, HPS-1 through HPS-10, are caused by mutations in human genes *HPS1* through *HPS10,* respectively. These HPS genes encode proteins that form Biogenesis of Lysosome-related Organelles Complexes (BLOCs), which are essential for the synthesis of LROs such as melanosomes, platelet dense bodies, lamellar bodies, and lytic granules of cytotoxic T lymphocytes (Dell’Angelica et al., 2000; Dell’Angelica, 2004). Defects in these LROs result in hypopigmentation and platelet storage deficiencies observed in all patients with HPS (Huizing et al., 2000). Three BLOCs-BLOC-1, BLOC-2, and BLOC-3-have been associated with the development of HPS. As displayed in Figure 1, BLOC-1 is a multimeric complex containing proteins HPS-7, HPS-8, and HPS-9, along with other subunits such as Snapin and other BLOS subunits (Li et al., 2003; Cullinane et al., 2012; Badolato et al., 2012; Starcevic and Dell’Angelica 2004). BLOC-2 is comprised of three large subunits, HPS-3, HPS-5 and HPS-6 (Carmona-Rivera et al., 2011; Starcevic and Dell’Angelica 2004; Bowman et al., 2019). BLOC-3 is a two-subunit complex composed of HPS-1 and HPS-4 proteins (Chiang et al., 2003). Mutations in adaptor protein complex-3 (AP-3)—a stable heterotetrametric complex (Figure 1) that assists in the transport of vesicles from endosomes and the biogenesis of LROs—have also been implicated in the development of HPS (Simpson et al., 1997). Specifically, genes *AP3B1* and *AP3D* encode the subunits β3A and δ of AP-3, respectively, and mutations in these genes result in HPS subtypes HPS-2 and HPS-10 (Simpson et al., 1997; Mohammed et al., 2019). Mutations found in the same BLOCs result in similar phenotypes with BLOC-3-related HPS subtypes having the most severe complications including the development of HPS-PF (Hermos et al., 2002; Anderson et al., 2003; Huizing et al., 2009). Individuals with a BLOC-1 and BLOC-2 related mutations present with milder OCA symptoms, and little to no pulmonary fibrosis when compared to individuals with mutations in *HPS1* and *HPS4* (BLOC-3 related) (Iwata et al., 2000; Anikster et al., 2001).

At present, there is still uncertainty about how mutations in the HPS genes lead to the clinical manifestations of HPS, but several studies have begun to identify the functions of HPS proteins and their associated BLOCs. BLOC-1, BLOC-2, and AP-3 interact and assist in melanosome biogenesis by trafficking necessary components from endosomes away from the degradative lysosomal pathway (Di Pietro et al., 2006; Setty et al., 2007; Truschel et al., 2009). BLOC-3 is a Rab32 and Rab38 (Rab32/38) guanine nucleotide exchange factor (GEF), which is capable of activating small GTPases and affecting downstream targets via intracellular signaling and protein trafficking. Rab32/38 assists in the trafficking of enzymes important for proper pigmentation, and mutations in Rab38 and the prenylation machinery that links Rab proteins to membranes can result in alteration of platelet and melanosome formation, causing pigmentation defects (Gerondopoulos et al., 2012; Vicary et al., 2016). Additionally, activated Rab32/38 is needed for the transport of tyrosinase and tyrosinase-related protein I (TYRP1) from premature endosomes to melanosomes in melanocytes (Wasmeier et al., 2006). Disruption of this Rab32/38 function leads to the common oculocutaneous manifestations found in individuals with HPS-1 (Ikawa et al., 2015). AT2 cells play a crucial role in surfactant synthesis and secretion in the lungs (Guttentag et al., 2005; Atochina-Vasserman et al., 2011). In AT2 cells, Rab38 assists in the maturation and maintenance of lamellar bodies, surfactant homeostasis, and the structure of the alveolar epithelium (Osanai et al., 2008; Zhang et al., 2011). AT2 cells lacking Rab38 have enlarged lamellar bodies with altered surfactant contents, leading to the progressive lung fibrosis seen in patients with subtypes HPS-1 and HPS-4. While studies suggest that mutations causing defects in BLOC-3 and Rab32 in macrophages and monocytes might lead to susceptibility to granulomatous colitis, little is still known about the molecular and cellular basis of this connection.

## Epidemiology

HPS has been reported in patients worldwide and seen in individuals with different ethnic backgrounds, including India, China, Japan, Western Europe, the Middle East, and Latin America (Carmona-Rivera et al., 2011). However, it has been most commonly reported in the Caribbean island of Puerto Rico. In the northwest region of Puerto Rico, 1 in 1800 suffer from subtype HPS-1, and 1 in 21 are a carrier of the founder mutation, a 16 base pair (bp) duplication in exon 15 of *HPS1* (El-Chemaly and Young, 2016). Molecular analysis of non-Puerto Rican Hispanic HPS patients have revealed that none of these patients carries the Puerto Rican mutation in the *HPS1* gene, but rather other mutations in the *HPS1*, *HPS4* and *HPS5* genes (Carmona-Rivera et al., 2011). In central Puerto Rico, 1 in 4000 people are affected by subtype HPS-3 (Anikster et al., 2001; Santiago Borrero et al., 2006). Ashkenazi Jews comprise a majority of the non-Puerto Rican patients affected by the HPS-3 subtype (Huizing et al., 2001). Given how the severity of HPS clinical symptoms can vary depending on the HPS subtype, it may be valuable to do wider screenings for HPS in patients with mild hypopigmentation disorders. One study has shown that 35% of the German albino population are candidates for having mild HPS (Passmore et al., 1999; Huizing et al., 2001).

## Clinical Manifestations

All HPS subtypes result in OCA and a platelet storage pool deficiency (Seward and Gahl, 2013). However, each subtype of HPS is distinguished from the others by signs, symptoms and genetic cause, as described in the above sections (Huizing et al., 1993; Li et al., 2004; Sánchez-Guiu et al., 2014). PF can occur in patients with mutations in *HPS1, AP3B1 (HPS2),* and *HPS4,* and individuals in the northwest region of Puerto Rico are largely affected (Huizing et al., 1993). Other subtypes like HPS-3, HPS-5, and HPS-6 have milder symptoms (Kelil et al., 2014; Vicary et al., 2016).

### Skin/Hair/Eye Hypopigmentation

Hair color in HPS patients ranges from white to brown and can also darken with age. Skin color can be white or olive (Huizing et al., 1993). Patients with hypopigmentation of the skin are at increased risk of solar keratosis, photo-aging of the skin, sunburn, and 3 major forms of cutaneous malignancy: squamous cell carcinoma, basal cell carcinoma, and melanoma (Huizing et al., 2000). The risk of UV-associated skin damage in patients with HPS is highest in childhood (Seward and Gahl, 2013). The eyes in almost all children with HPS have nystagmus and periodic alternating nystagmus which cause wandering eye movements and lack of visual attention (Gradstein et al., 2005).

### Bleeding Diathesis

The HPS platelet storage pool deficiency causes bleeding diathesis and often manifests in infancy and persists throughout life. Bleeding can occur with simple trauma to the skin, dental extractions or dental cleanings, and menstrual bleeding for many women with HPS (Seward and Gahl, 2013). Pregnancies have to be managed as high risk because of the bleeding diathesis (Beesley et al., 2008). Epistaxis usually occurs in childhood and diminishes after adolescence (Huizing et al., 1993). Patients with HPS wear medical alert bracelets: due to bleeding diathesis complications, blood-thinning medications such as ibuprofen, aspirin, and warfarin should be avoided. Instead, desmopressin can be used to prevent these bleeding complications. Additionally, platelet transfusions may be required in the case of severe bleeding episodes or surgical procedures (El-Chemaly and Young, 2016).

### Colitis

The granulomatous inflammation in the bowel of patients with HPS resembles that of Crohn’s disease both clinically and pathologically (Salvaggio et al., 2014). The involvement of the gastrointestinal tract by a granulomatous colitis has been described in patients with HPS-1, HPS-4 and HPS-6 (Huizing et al., 1993; El-Chemaly and Young, 2016). Severe colitis affects approximately 15% of patients with HPS (El-Chemaly and Young, 2016). Treatment of HPS-related colitis entails a similar treatment to Crohn’s disease with anti-inflammatory drugs, immunosuppressants, and infliximab (Hazzan et al., 2006; Hussain et al., 2006). Surgery is the last resort for patients with further complications (Seward and Gahl, 2013).

### Immunodeficiency

Individuals with HPS-2, which is characterized by the lack of the β3A subunit in the Adaptor protein-3 (AP-3), are immunodeficient (Fontana et al., 2006). Neutropenia, an abnormally low neutrophil count, has been associated with AP-3-deficient HPS-2 patients, including the individuals with the pathogenic variants in AP3B1 (Huizing et al., 1993; Fontana et al., 2006). The mechanisms that contribute to the defective immune system in HPS-2 patients remain unknown. Studies using cells from HPS-2 patients predict cellular processes for the immune dysfunction are associated with impaired function of cytotoxic T cells and Natural Killers (NK) cells (Gil-Krzewska et al., 2017). Dendritic cells from HPS-2 patients showed severely impaired cytokine and chemokine release, indicating that faulty cytokine secretion could be one of the major factors contributing to immunological deficiency in individuals with HPS-2 (Prandini et al., 2016; Gil-Krzewska et al., 2017). It is interesting to note that, in addition to HPS-2, NK cells recovered from HPS-1 individuals also had reduced cytotoxicity and lytic functions. HPS-1 patients are generally not immunodeficient and are not predisposed to infections because the reduced NK cell activity was mitigated with increased cell number (Gil-Krzewska et al., 2017). However, the authors found normal NK cell activity in HPS-4 individuals, suggesting that BLOC-3 complex does not have a direct role in regulating NK cell cytotoxicity. Animal models of HPS have provided supportive evidence for defective immune system associated with HPS-2. Using cells from *AP3B1(HPS2)* deficient mice, Sasai et al. demonstrated that AP-3 is responsible for the trafficking of TLR9 to this subcellular compartment, contributing to pattern recognition of viral nucleic acids (Sasai et al., 2010).

### Pulmonary Fibrosis

Patients with HPS-1, HPS-2, and HPS-4 most commonly experience associated PF. HPS-PF and IPF are considered similar diseases because they show similar patterns clinically and histologically (Vicary et al., 2016). In patients with HPS-1, about 100% of individuals develop HPS-PF. Similar to IPF, HPS-PF is characterized by a progressive fibrogenesis of the lung parenchyma and interalveolar septa that eventually leads to death from respiratory failure (Seward and Gahl, 2013). Both forms of PF also manifest similar symptoms, including dyspnea and incrementing debilitating hypoxemia. One difference is that IPF typically manifests in individuals over age 50 years old while HPS-PF typically manifests in individuals at the age of 30–40 years old. Additionally, the average survival time after diagnosis with IPF and HPS is about 3 years (Witkop et al., 1990; Vicary et al., 2016; Raghu et al., 2018). To date, the pathogenesis of PF in HPS remains unknown, and the PF is the leading cause of death in HPS patients. For the remaining of this article, we focus on the clinical management strategies of HPS-PF, as well as clinical and experimental evidences using cell culture and animal models investigating the pathogenesis of HPS-PF.

## Clinical Managements of HPS-PF

### Diagnosis

The diagnosis of pulmonary fibrosis is performed with a high-resolution computed tomography of the chest (HRCT). HRCT is performed using a CT scanner that takes thin-slice chest images with lung details. Some of the findings in the HRCT in the early stages include septal thickening, ground-glass pattern, mild reticulation, and in the advanced stages of HPS-PF, severe reticulation, bronchiectasis, subpleural cysts, and peribronchovascular thickening may be found. HRCT is more sensitive than chest radiography in the evaluation of the progression of HPS-PF, and provides a good radiologic monitoring of disease progression that correlates well with age, extent of pulmonary dysfunction, and genetic findings (Avila et al., 2002).

### Pirfenidone as the Treatment for HPS-PF

Although the mechanism of action of pirfenidone is still unknown, it has been shown to have both anti-inflammatory and anti-fibrotic effects (Cho and Kopp, 2010). Inhibition of both production and activity of TGF-β is considered as a key characteristic of the anti-fibrotic mechanism of pirfenidone (Myllärniemi and Kaarteenaho, 2015). Pirfenidone treatment has been shown to drastically suppresses the TGF-β gene transcription by 33% in bleomycin-induced lung injury hamster model (Iyer et al., 1999b) and in the other study pirfenidone extinguishes bleomycin-induced overexpression of procollagen I and III genes (Iyer et al., 1999a). It was subsequently approved by the United States Food and Drug Administration as treatment for IPF in 2014, after a multinational phase 3 trial conducted for 52 weeks which showed that pirfenidone reduced disease progression in patients with IPF and was associated with acceptable side effects and fewer deaths (King et al., 2014; Meyer and Decker, 2017). Pirfenidone was investigated at the National Institutes of Health (NIH) Clinical Center as a treatment for HPS-PF (O’Brien et al., 2011; O’Brien et al., 2018; Gahl et al., 2002). An initial trial investigated the drug for mild to moderate HPS-PF patients. However, the study was terminated due to futility (Gahl et al., 2002). A subsequent study showed that subjects with an initial forced vital capacity (FVC) between 50 and 75% who received pirfenidone lost forced vital capacity at a slower rate than those in the placebo group. Yet, the entire data showed no significant difference between the pirfenidone and placebo groups (Gahl et al., 2002; O’Brien et al., 2018). The results of these trials are inconclusive if pirfenidone is a beneficial treatment for HPS-PF patients. While this study did not provide sufficient data whether pirfenidone is advantageous in treatment for HPS-PF, it offered evidence about the safety of pirfenidone in patient with mild to moderate HPS-PF. Patients experienced comparatively few and mild side effects such as photosensitivity rash which was treatable and conceivably an elevated creatine phosphokinase. Continued long-term study follow-up is important to inform clinical practice and to demonstrate the efficacy and safety of pirfenidone, as pulmonary fibrosis patents are expected to receive prolonged treatment with pirfenidone. Accordingly, a more recent study followed three HPS-PF patients with open-label pirfenidone for 12.8, 8.4, or 18.1 years (mean of 13.1± 2.8 years), and twenty-one historical controls randomized to placebo (O’Brien et al., 2018). Changes in the rate of decline of FVC and the diffusing capacity for carbon monoxide (DLCO) in response to prolonged treatment with pirfenidone was various in these 3 patients. Overall, long-term pirfenidone treatment demonstrated positive improvement in the results of serial pulmonary function tests and HRCT scans in 2 out of 3 patients. Moreover, all patients who shifted from placebo to open-label pirfenidone experienced positive changes in the rate of FVC and DLCO. In terms of pirfenidone treatment safety, all 3 patients had normal levels of aspartate aminotransferase and normal blood test results at their final evaluation except for low serum potassium level and high platelet count in 2 different patients. The results of this study demonstrated favorable clinical outcome with few manageable adverse effects on HPS-PF patients that were treated with pirfenidone for several years. These results suggest that the drug can be considered on a case-by-case basis for HPS-PF patients. Multiple clinical trials are investigating single drug therapy for pulmonary fibrosis and have been unsuccessful. For that reason, pulmonary fibrosis may need to be treated with a multidrug regimen to target cellular and molecular pathways that contribute to fibrosis.

### Lung Transplantation

Lung transplantation remains the only available therapy for patients with HPS-PF. HPS-PF patients should be referred for lung transplant evaluation in the early stages of the disease (El-Chemaly et al., 2018a). The bleeding diathesis associated with HPS is not a major impediment to perform surgery in HPS patients, although it can be a potential contraindication because of the tendency to bleed due to deficiency of platelet dense bodies (El-Chemaly et al., 2018b). Successful lung transplants have been performed in individuals with HPS-1 despite the risks of bleeding (El-Chemaly and Young, 2016). The bleeding diathesis is usually treated with desmopressin or platelet transfusions. In Puerto Rico, centers with capabilities in lung transplantation are not available. For this reason, connections with centers for lung transplantation in the United States are important because this is the only available therapy for patients with HPS-PF (Vicary et al., 2016).

## Pathogenesis of Pulmonary Fibrosis

The pathogenesis of HSP-PF is unknown. To date, there are promising clinical studies investigating the pathogenesis of PF in HPS patients. However, these studies are limited by the availability of HPS lung tissue due to the disease rarity and the dangers of lung transplantation caused by bleeding diathesis (El-Chemaly et al., 2018a). For that reason, researchers have used murine models since they share many aspects with the human disease. In both human and mice, the disease affects the biosynthesis of the related organelles: melanosomes, lysosomes, and platelet dense granules (Swank et al., 2000). Thus, researchers can easily assess cells and tissues from various HPS mouse models, and perform genetic and pathological manipulations. In addition to a limited number of clinical studies profiling immune cell dysregulation in HPS patients, current understandings of the pathogenesis of HPS-PF are largely based on examining cellular and molecular pathways involved using HPS mice and bleomycin-induced lung fibrosis as experimental models.

### Cellular Pathways

#### Epithelial Cell Stress and Apoptosis

**FIGURE 2:**
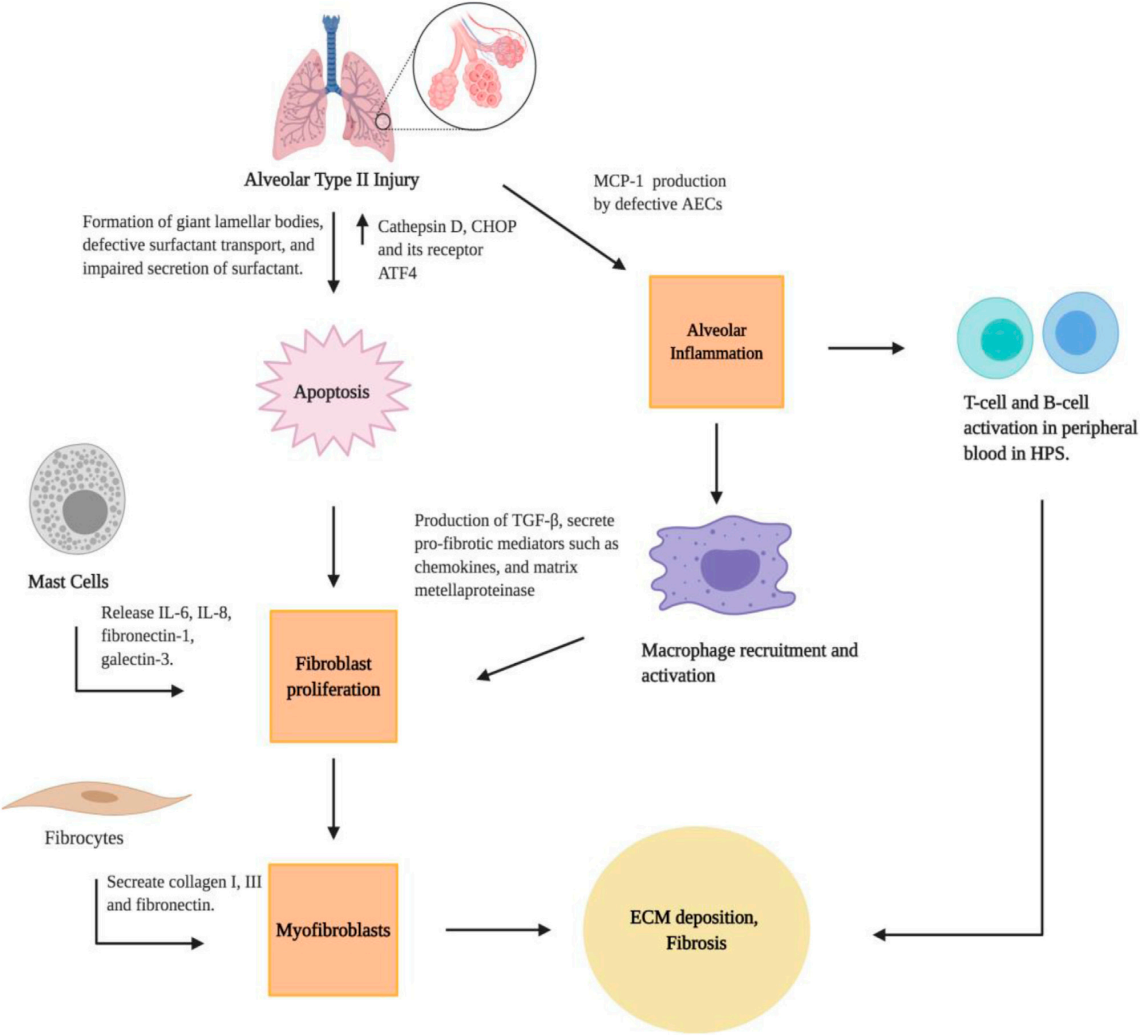
Cellular pathways for the development of lung fibrosis in HPS. Created with BioRender.com.

The pulmonary alveolar epithelium is mainly composed of two types of epithelial cells: alveolar type I (AT1) and AT2 cells. AT1 cells are large squamous cells that cover 95% of the alveolar surface area and are essential for the air-blood barrier functions of lungs. AT2 cells are smaller and cuboidal cells known for their functions in synthesizing and secreting pulmonary surfactant (Wang et al., 2018). Murine models and lung pathology specimens have provided insights into the role of lung epithelium in the pathogenesis of PF. In HPS patients, features of HPS-PF include the apoptosis and dysfunction of AT2 epithelial cells (Figure 2), which appear foamy because of the formation of giant lamellar bodies (Nakatani et al., 2000). Studies have shown that *pale-ear* mice (with spontaneous *HPS1* mutation) develop giant lamellar bodies in AT2 epithelial cells at baseline and after bleomycin challenge (Tang et al., 2005; Young et al., 2007). Additionally, investigations using *HPS1/2* double mutant mice demonstrated abnormal intracellular accumulation of surfactant proteins in AT2 cells. Such surfactant accumulation can lead to the production of Cathepsin D, a lysosomal stress protease, and subsequent expression of the pro-apoptotic endoplasmic reticulum (ER) stress factor CHOP and its transcription factor ATF4, to induce apoptosis. The combined lysosomal and ER stress in AT2 epithelial cells results in significant AT2 epithelial cell apoptosis, airspace enlargement, fibroblast proliferation, and spontaneous lung fibrosis in *HPS1/2* double mutant mice (Mahavadi et al., 2010). In fact, Bone marrow transplantation experiments demonstrate that, in various HPS mouse models, susceptibility to bleomycin-induced fibrosis is determined by the threshold of AT2 epithelial cell apoptosis, indicating the critical role for lung epithelial cells in the regulation of immune activation and subsequent fibroproliferative remodeling processes (Zoz et al., 2011; Young et al., 2012).

#### Alveolar Epithelial Cell/Macrophage Interaction

The mechanisms linking AEC dysfunction and fibrotic remodeling, specifically the interactions between epithelial dysfunction, alveolar macrophage activation, and ultimately fibroblast proliferation and differentiation, are understudied in the context of HPS-PF (Zoz et al., 2011; Young et al., 2012). There is strong evidence that macrophage-mediated inflammation contributes to the development of PF in HPS in patients (Rouhani et al., 2009). The authors found a significantly higher concentration of total bronchoalveolar lavage cells and alveolar macrophages in HPS-PF patients. The alveolar macrophage activation and lung inflammation in HPS patients are associated with high lung concentrations of cytokines and chemokines such as monocyte chemoattractant protein-1 (MCP-1), macrophage inflammatory protein-1α, and granulocyte-macrophage colony-stimulation factor) (Rouhani et al., 2009). This provide evidence that alveolar macrophage dysfuction may contribute to the PF in HPS-1 patients. Using the *pearl-ear* mouse model of HPS-2, Young et al. found HPS alveolar macrophages are hyperresponsive to TNF-α and LPS stimulation (Young et al., 2006). In a follow-up study, the authors found that an increase of macrophages in the lungs of HPS mice was associated with excessive MCP-1 production from AECs, and that blocking MCP-1/CCR2 signaling eliminated the increased macrophage recruitment in the lung while also reducing excess fibrotic responses. Lung macrophages activated by MCP-1 produce TGF-β, which promotes fibrosis through activation and differentiation of fibroblast cells (Young et al., 2016). These studies highlight that increased MCP-1 production by dysfunctional AECs results in recruitment and activation of TGF-β-producing macrophages, and epithelial-macrophage interactions stimulate fibrotic remodeling.

#### Lymphocytes

In addition to alveolar macrophages, lymphocyte populations may also contribute to the overall increased total bronchoalveolar lavage cell in HPS-1 patients (Rouhani et al., 2009). Studies using peripheral blood samples from HPS patients have shown that levels of CD38 ^+^ memory CD27-B cells, IgA + memory CD27^+^ B-cells, IgM+ and IgD + B cells, and CD39 ^+^ T helper cells were increased, and that CD39-T helper cells was reduced in HPS-PF when compared with unaffected controls (El-Chemaly et al., 2018a). Interesting, B cell abnormalities have been identified in other fibrotic diseases such as IPF, which has been found to be related to disease progression (Xue et al., 2013). In HPS-PF, it was discovered that high peripheral blood concentrations of activated T-cell and B-cell populations are associated with altered leptin and inflammatory cytokine levels (Figure 2; El-Chemaly et al., 2018a). Leptin can activate human B cells to induce synthesis and secretion of cytokines that are critical in the regulation of immune activation, such as IL-6, IL-10, and TNF-α (Agrawal et al., 2011). Leptin can also stimulate the proliferation and activation of both CD4 and CD8 T cells (Martin-Romero et al., 2000). Taken together, these studies suggest that the activation of T-cells and B-cells is a critical feature of HPS-PF. Additional studies in animal models are required to investigate the role of leptin in the regulation of lymphocyte activation in the pathogenesis of HPS-PF.

#### Mast Cells

Mast cells have been known to be present in patients with PF and they present signs of on-going degranulation (Kawanami et al., 1979). Recent studies suggest that mast cells may drive fibrotic responses to lung injury by stimulating fibroblasts proliferation and ECM production in IPF (Wygrecka et al., 2013). In HPS-1 patients, *in situ* study showed that HPS-1 mast cells contained abnormalities in mast cell granules, which are also classified as LROs (Kirshenbaum et al., 2016). *In vitro* experiments of derived HPS mast cells showed a reduction of CD117 and FcεRI expression, and increased expression of CD63 and CD203c. A reduction of granule formation was verified in cell line derived from one HPS-1 patient, along with increased release of IL-6, IL-8, fibronectin-1 and Gal-3 (Kirshenbaum et al., 2016). Interestingly, these proteins are known to participate in HPS-PF and are also produced by fibroblast (Cullinane et al., 2014) and endothelial cells (Kusuma et al.. 2012). The results showed that HPS-1 mutated mast cells have abnormal granule formation, cell activation, release of cytokines, and potentially affect synthesis of matrix deposition. Similar to the lymphocyte population, future work in animal models are required to establish a direct role of mast cells in the pathogenesis of HPS-PF.

#### Fibrocytes

Fibrocytes are circulating bone marrow-derived progenitor cells, and are of interest for the study of fibrotic disorders. Fibrocytes are positive for CD45, and express extracellular matrix proteins such as vimentin, fibronectin, and collagen I and III (Trimble et al., 2014). They are found in injured tissues, and can serve as an important source of myofibroblasts (Keeley et al., 2010). Studies have demonstrated the presence of an increased number of fibrocytes in the circulation of IPF patients that are known to have fibroblast activation and macrophage inflammation (Reilkoff et al., 2011). Expanded pool of fibrocytes are found in the peripheral blood of IPF patients (Mehrad et al., 2007; Andersson-Sjoland et al., 2008) and is an independent predictor of mortality (Moeller et al., 2009). In individuals with HPS, levels of circulating CXCR4-positive fibrocytes in peripheral blood were markedly elevated in comparison with subjects without lung disease and normal controls. Longitudinally, these elevations correlated with subsequent death from progressive lung disease (Trimble et al., 2014). The results suggest that circulating fibrocytes may be an important source of myofibroblasts, and a potential biomarker of prognosis in HPS-PF.

### Molecular Pathways

#### Chitinase-3-like-1 and its Receptors

**FIGURE 3:**
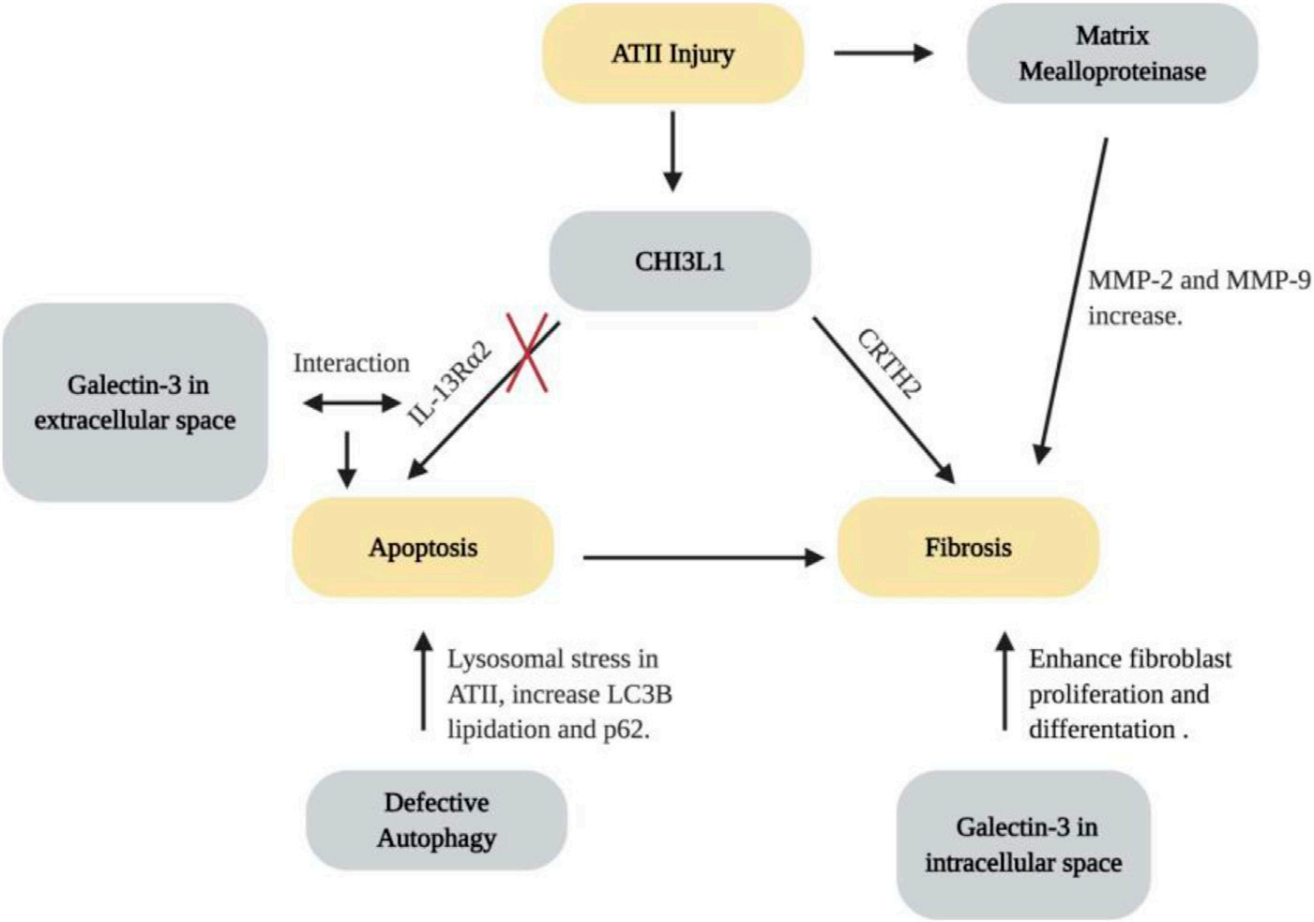
Molecular pathways for the development of lung fibrosis in HPS. Created with BioRender.com.

The glycoside hydrolase 18 (GH18) family is an ancient gene family which contains true chitinases that enzymatically cleave chitin and chitinase-like proteins. GH18 is widely expressed in archea, prokaryotes and eukaryotes (Funkhouser and Aronson, 2007). In mammals, endogenous chitin does not exist. However, chitinases and chitiase-like proteins (including CHI3L1) are expressed at high levels in the lungs (Bussink et al., 2007; Zhou et al., 2014). The dysregulation of CHI3L1 is associated with the development, severity or progression of many pulmonary diseases, including asthma, COPD and IPF (Sohn et al., 2010; Matsuura et al., 2011; Sakazaki et al., 2011; Zhou et al., 2014; Kang et al., 2015). It is believed that CHI3L1 is a protein that plays a protective role in the lung by decreasing cell death and stimulating fibroproliferative repair (Zhou et al., 2014). In HPS, levels of CHI3L1 are higher in patients with HPS-PF in comparison with patients without pulmonary fibrosis, where higher levels are associated with greater disease severity. Using murine models, Zhou et al. found that the animals with BLOC-3 mutation have a defect in the ability of CHI3L1 to restrain epithelial cell death, yet CHI3L1 exhibits exaggerated fibroproliferative effects, promoting fibrosis by inducing alternative macrophage activation and fibroblast proliferation (Zhou et al., 2015). The two distinctive features of CHI3L1 are mediated by trafficking of two CHI3LI receptors, IL-13Rα2 and CRTH2. The increase of apoptosis results from the abnormal localization of IL-13Rα2, which is caused by the dysfunction of BLOC-3. Fibrotic effects were caused by interactions between CHI3L1 and CRTH2 receptors which traffic normally (Zhou et al., 2015). These studies suggest that CHI3L1 and its receptors are dysregulated and play critical roles in the generation and progression of lung fibrosis associated with HPS (Figure 3). In addition, these responses are largely mediated by CRTH2, which may serve as a therapeutic target. Multiple clinical trials were designed to assess the effects of CRTH2 antagonism on asthma control. Future studies will be required to explore the possibility of repurposing these small molecular CRTH2 antagonists for HPS-PF treatment.

#### Galectin-3 Dysfunction and its Interaction with CHI3LI

Gal-3 is a β-galactoside–binding lectin with pro-fibrotic effects (Young et al., 2006; Cullinane et al., 2014; Li et al., 2014). Gal-3 inhibitor TD139 is safe and well-tolerated, and has been shown to decrease plasma biomarkers associated with IPF progression in a phase I/IIa trial (Hirani et al., 2020). Strong evidence has indicated the role of Gal-3 in the development of HPS-PF. In samples from HPS-1 patients, AT2 cells, alveolar macrophages, and fibroblasts have high levels of Gal-3 expression and intracellular accumulation. It is speculated that the accumulation of Gal-3 in the cells of HPS individuals can be explained by the abnormal trafficking in the endosomal recycling compartment, which can contribute to fibrogenesis in HPS-PF (Cullinane et al., 2014). Consistently, murine studies have found that Gal-3 has increased levels in the extracellular space, traffics abnormally, and accumulates in lung fibroblasts and macrophages. Extracellular Gal-3 stimulates epithelial apoptosis and intracellular Gal-3 enhances fibroblast survival and proliferation as well as myofibroblast and macrophage differentiation. Further studies demonstrate that Gal-3 interfere with CHI3L1 signaling by competing for IL-13Rα2 binding. As a result, Gal-3 diminishes the anti-apoptotic effects of CHI3L1 in epithelial cells while increasing macrophage Wnt/ß-catenin signaling (Zhou et al., 2018). Therefore, Gal-3 contributes to the exaggerated injury and fibroproliferative repair response by altering the anti-apoptotic and fibroproliferative effects of CHI3L1 and its receptors (Figure 3). It can be speculated that Gal-3-based therapies may very well act in an additive or synergistic manner with interventions that augment membrane expression of IL-13Rα2 or block CRTH2. Additional investigations will be required to assess the utility of each of these approaches.

#### Matrix Metalloproteinases

MMPs are a family of zinc-dependent proteolytic enzymes known for their role in degrading extracellular matrix proteins and activating or inhibiting other effector molecules (Summer et al., 2019; Parks and Shapiro 2001; Greenlee et al., 2007). Activities of various MMPs are known to be dysregulated and linked to the pathogenesis of numerous chronic lung diseases, including asthma, emphysema, cystic fibrosis and IPF (Summer et al., 2019; Cataldo et al., 2000; Chelladurai et al., 2012; Craig et al., 2015; Henry et al., 2002). Recent studies have shown an increase in enzymatic activity of MMP-2 and MMP-9 in lungs of pearl ear HPS-2 mice after bleomycin challenge. Likewise, even at baseline, the amount and level of activity of different MMPs are increased in the lungs and bronchoalveolar fluid of mice carrying the BLOC-3 gene mutations (Figure 3; Summer et al., 2019). However, although MMP activity appears to be increased in the lung of HPS patients, a correlation between MMP activity and disease severity was not observed. More studies investigating the dysregulation of MMPs are necessary to better understand their contribution to the progression of HPS lung disease.

#### Autophagy

Autophagy is a basic homeostatic mechanism through which a cell can degrade and recycle unnecessary or damaged proteins and organelles via its lysosomes (Eskelinen 2008; Ahuja et al., 2016). Autophagy can be categorized into macroautophagy, microautophagy, and chaperone-mediated autophagy (Mizushima 2007). Macroautophagy is a process involving rearrangement of subcellular membranes to isolate cytoplasm and organelles for delivery to the lysosomal compartment. In HPS-1, melanosome-targeted proteins are localized to membranous complexes. These membranous complexes have similarities to macroautophagosomes, and studies have demonstrated that the membranous complexes of HPS-1 melanocytes are macroautophagosomal representatives of the lysosomal compartment (Smith et al., 2005). Additionally, dysfunctional autophagy has been known to play an important role in the development of numerous pathologies, including lysosomal storage diseases, neurodegenerative diseases and organ-specific diseases, including lung fibrosis (Cataldo et al., 2000; Cao et al., 2006; Komatsu et al., 2006; Pacheco et al., 2007; Settembre et al., 2008; Araya et al., 2013). Recent studies have demonstrated that defective autophagy might result in the excessive lysosomal stress in HPS. Key autophagy proteins, including lipidated LC3B proteins and p62, were increased in *HPS1/2* double mutant murine models (Ahuja et al., 2016). LC3B preferentially binds to the interior of lamellar bodies in the AT2 epithelial cells of HPS murine models, but not on the membrane of lamellar bodies, leading to deficient autophagy and pro-apoptotic caspase activation (Ahuja et al., 2016). These studies added to the body of literature on AT2 cell apoptosis that loss of HPS-1 protein results in impaired autophagy, which contributes to lamellar body degeneration and AT2 epithelial apoptosis, and defective autophagy might therefore play a critical role in the initiation and development of HPS-PF (Figure 3).

## New Models and Approaches to Study HPS-PF

### Lentiviral-Mediated Gene Transfer

Therapies targeting the cellular and molecular pathways in the diseases are usually life-long treatments. For autosomal recessive genetic diseases, efforts to develop gene therapy began soon after the genetic mutations are discovered. For example, patients with Cystic Fibrosis were treated with an adenoviral vector carrying a *CFTR* expression cassette (Zabner et al., 1993). Lentiviral vectors are known to be able to integrate into the host genome while display low immunogenicity, ensuring persistent gene correction and safety (Sinn et al., 2008; Stocker et al., 2009). Thus, genetic correction of HPS mutations using lentiviral approaches can provide an alternative therapeutic option. Studies have demonstrated that lentiviral-mediated gene transfer corrects expression of the *HPS1* gene in melanocytes, restores BLOC-3 function, and corrects pigmentation in these cells (Ikawa et al., 2015). The development of lentiviral vectors which transduce lung tissue efficiently have opened up room for development of gene therapy for HPS-PF and other clinical manifestations of HPS in general. However, how to target lung AT2 epithelial cells specifically and efficiently in the lung requires future research before gene therapy can be considered for potential use in correcting BLOC-3 mutations in patients.

### Pluripotent Stem Cell-Derived Alveolar Organoids and 3D Models

Generating *in vitro* models of AT2 cells to study lung diseases has been difficult due to the poor accessibility and the difficulty of isolating and culturing primary AT2 cells. For that reason, methods of generating and expanding AT2 cell organoids from iPSCs have been established and utilized as a model for various lung diseases (Gotoh et al., 2014; Yamamoto et al., 2017). Using similar approaches, a recent study has created disease-specific iPSCs and gene-corrected counterparts from a HPS-2 patient. Live cell imaging showed altered distribution of lamellar bodies with enlargement, and impaired surfactant protein secretion in HPS-2-iPSC-derived AT2 cells (Korogi et al., 2019). These findings demonstrate the benefits of using human iPSC-derived AT2 cellular models for future research on the alveolar lung diseases.

Additional efforts have been devoted to the development of iPSC-derived three dimensions (3D) multi-cellular organoid models. Chen et al. were able to develop lung bud organoids (LBOs) that contain multiple cell types and develop into branching airway and early alveolar structures similar to developing lung buds *in vivo* (Chen et al., 2017). The authors then introduced various HPS mutations, and found fibrotic changes characterized by cells with increased expression of collagen genes, fibronectin, and mesenchymal markers (Chen et al., 2017; Strikoudis et al., 2019). Genome-wide expression analysis revealed an upregulation of interleukin-11 (IL-11) in the epithelial cells of HPS mutant fibrotic organoids. Additionally, IL-11 induced fibrosis in wildtype (WT) organoids, while its deletion prevented fibrosis in *HPS4* mutated organoids, suggesting IL-11 as a potential therapeutic target (Strikoudis et al., 2019). Human pluripotent stem cell (hPSC)-derived 3D lung organoids have been shown to be a variable resource in modeling fibrotic lung disease that assembles human disease features, and is an innovative strategy allowing the identification of potential novel therapeutic targets.

### CRISPR-Cas9 Approaches for Gene Editing

Studies have shown that CRISPR/Cas9 gene editing can generate small mutations in a site-directed manner leading to permanent gene inactivation in a variety of cell types (Doudna and Charpentier, 2014). In HPS, as discussed in previous sections, AT2 epithelial cells in BLOC-3 and AP-3-related HPS subtypes are believed to be dysfunctional, and the sensitivity of AT2 epithelial cells to stress-induced apoptosis may determine subsequent susceptibility to lung fibrosis. Using a mouse AT2 cell line MLE-15, Kook et al. succesfully used CRISPR/Cas9 gene editing approaches to generate a series of cell lines bearing HPS-specific mutations. The authors found increased expression of MCP-1, previously reported as the central mediator of macrophage activation in HPS patients and mouse models, in MLE-15/HPS-1 and MLE-15/HPS-2 cell lines. It is interesting to note that higher MCP-1 expression was also found in MLE-15/HPS-9 cells, suggesting BLOC-1 mutations may also cause inflammatory macrophage infiltration (Kook et al., 2018). The MLE-15/HPS cells replicate known characteristics of primary HPS AT2 epithelial cells, providing an alternative and permanent platform for the studies of AT2 cellular pathophysiology that could accelerate progress toward developing novel therapies.

In addition to the nonhomologous end joining mechanism that CRISPR-Cas9 system uses to generate microinsertions or microdeletions, it has also been programmed to correct disease-causing genetic mutations. A recent study shows that microduplications, such as that found in HPS-1 patients of Peurto Rico origins, can be efficiently corrected simply by generating a double-strand break (DSB). Streptococcus pyogenes Cas9 (SpyCas9) was used to generate a DSB in HPS-1 patient-derived B lymphocytes near the center of the 16 bp duplication that is responsible for the mutation. It is discovered that efficient genotypic correction was observed by the microhomology-mediated end joining pathway (Iyer et al., 2019). This approach has provided proof-of-concept for the use of gene editing in future HPS treatment.

## Conclusion

Although the direct link between HPS gene mutations and lung pathobiology is unclear at present, significant advances in identifying the cellular and molecular pathways affected by HPS genetic mutations have been identified. AT2 epithelial cell apoptosis and dysfunction, which may be attributed to giant lamellar body formation, surfactant accumulation, and severe lysosomal and ER stress, are believed to be the initiating step for epithelial cell injury and subsequent lung fibrosis. Immune cell activation such as T and B cells, mast cells, and macrophages can promote fibroblast accumulation and myofibroblast differentiation that are responsible for the excessive deposition in extracellular matrices and lung architecture destruction (King et al., 2011). In lung fibrosis, myofibroblasts may also be derived from circulating fibrocytes originated from bone marrow progenitors, from transdifferentiation of epithelial cells through epithelial-mesenchymal transition (EMT) or from transdifferentiation of endothelial cells through endothelial mesenchymal transition (EndoMT) (Phan 2012). Further studies will be required to evaluate the possible involvements of EMT and EndoMT as sources of myofibroblasts in HPS-PF (Franks et al., 2008; Hashimoto et al., 2010).

*In vitro* studies of tissues, peripheral blood cells, and bronchoalveolar lavage fluid from patients along with *in vivo* studies using murine models have been useful for studying the potentially intervenable biological pathways of HPS. Research on the involvement of CHI3L1 and its receptors, its interaction with Gal-3, and the activation of MMPs, as well as defective autophagy pathways have shown promising preliminary results that guide the progress needed to identify biomarkers and therapeutic targets. Gene transfer and editing approaches have potential to be a major alternative therapeutic strategy, but delivering the genes specifically and efficiently to lung epithelium remains the biggest hurdle. Future research is required to evaluate emerging cellular and molecular mechanisms in the development of HPS-PF. Extracellular vesicles (EVs) released by alveolar epithelial cells, lung fibroblast and endothelial cells are indicated in the pathogenesis of IPF (Bagnato and Harari, 2015; Bartel et al., 2020; Ibrahim et al., 2021). They drive lung fibroproliferative processes through activation of pro-fibrotic signaling pathways such as TGF-β signaling, Wnt and cellular senescence (Schafer et al., 2017; Martin-Medina et al., 2018; Barnes et al., 2019; Chanda et al., 2019; Parimon et al., 2019; Reyfman et al., 2019; Adams et al., 2020). Novel cellular models such as alveolar organoid and gene-edited epithelial cells will be valuable resources to examine the role of EVs in HPS-PF.

## Abbreviations

HPS, Hermansky-Pudlak Syndrome; PF, Pulmonary Fibrosis; OCA, oculocutaneous albinism; iPSCs, induced pluripotent stem cells; LRO, lysosome-related organelles; IPF, idiopathic pulmonary fibrosis; AT2, type II alveolar cells; CHI3L1, chitinase 3-like 1; Gal-3, Galectin-3; MMPs, matrix matalloproteinases; BLOCs, Biogenesis of Lysosome-related Organelles Complexes; GEF, guanine nucleotide exchange factor; TYRP1, tyrosinase-related protein I; AP-3, Adaptor protein-3; FVC, forced vital capacity; DLCO, diffusing capacity for carbon monoxide; HRCT, high-resolution computed tomography of the chest; MCP-1, monocyte chemotactic protein-1; HuMC, Human Mast cell.
